# A Feature Optimization Approach Based on Inter-Class and Intra-Class Distance for Ship Type Classification

**DOI:** 10.3390/s20185429

**Published:** 2020-09-22

**Authors:** Chen Li, Ziyuan Liu, Jiawei Ren, Wenchao Wang, Ji Xu

**Affiliations:** 1 Key laboratory of Speech Acoustics and Content Understanding, Institute of Acoustics, Chinese Academy of Sciences, Beijing 100190, China; lichen@hccl.ioa.ac.cn (C.L.); liuziyuan@hccl.ioa.ac.cn (Z.L.); renjiawei@hccl.ioa.ac.cn (J.R.); wangwenchao@hccl.ioa.ac.cn (W.W.); 2 University of Chinese Academy of Sciences, Beijing 100049, China

**Keywords:** underwater acoustic, ship radiated noise, feature optimization, joint training

## Abstract

Deep learning based methods have achieved state-of-the-art results on the task of ship type classification. However, most existing ship type classification algorithms take time–frequency (TF) features as input, the underlying discriminative information of these features has not been explored thoroughly. This paper proposes a novel feature optimization method which is designed to minimize an objective function aimed at increasing inter-class and reducing intra-class feature distance for ship type classification. The objective function we design is able to learn a center for each class and make samples from the same class closer to the corresponding center. This ensures that the features maximize underlying discriminative information involved in the data, particularly for some targets that usually confused by the conventional manual designed feature. Results on the dataset from a real environment show that the proposed feature optimization approach outperforms traditional TF features.

## 1. Introduction

Underwater acoustic target recognition based on radiated noise is one of the main applications of a passive sonar system. Based on the complexity of sound propagation in shallow sea and high background noise in a sea area, the sonar sensors may receive quite different audio signals coming from the same target. In the last decades, the traditional statistical feature like LoFar [1] and DEMON [2] have dominated many application scenarios of underwater acoustic target recognition until the development of Deep Neural Networks (DNN).

DNN have received extensive attention in underwater tasks in the past few years. Especially, several new attempts have been made to this field in order to improve ship type classification capability automatically in an unattended scene. The improvement of the system is mainly in two aspects: feature extraction and classifier training. In [3], wave structure was utilized straightly with support vector machines. After that, a deep learning model was used for feature extraction automatically [4] and complex classification model in underwater acoustic targets recognition. A time-delay neural network (TDNN) and convolutional neural network (CNN) were introduced for underwater acoustic targets recognition in [5,6] respectively. However, most deep learning-based approaches focused on utilizing the TF feature for deep learning classification models of ship data, ignoring the importance of extracting stable feature of targets. Especially due to changes in the underwater environment, the received signal may mislead the judgement from well-trained sonarmen. Hence, how to improve the discrimination ability of signal characteristics has attracted many researchers’ attention.

In contrast to most existing algorithms [7,8,9,10], in this paper, we try to improve the discriminative capability of features by introducing the idea of feature optimization into the underwater acoustic targets, firstly embedding the extractor. A feature optimization framework, including model structure and training strategy is proposed to enhance the discriminative capability of features. Some training criteria (loss functions) are utilized to constrain the distance between categories in training process, like center loss [11] and triplet loss [12]. The final objective function is focused on increasing inter-class and reducing intra-class distance. The result shows that the proposed feature optimization approach outperforms traditional TF features using DNN classifier with the same structure.

The rest of the paper is organized as follows. Section 2 gives an introduction of the auditory inspired deep neural network for ship type classification. The optimization framework we proposed is presented in Section 3. Section 4 compares the experimental results with traditional method at same dataset and describes the effect of feature optimization by feature visualization, and Section 5 concludes this work.

## 2. Architecture of Deep Neural Network for Ship Classification

**Figure 1:**
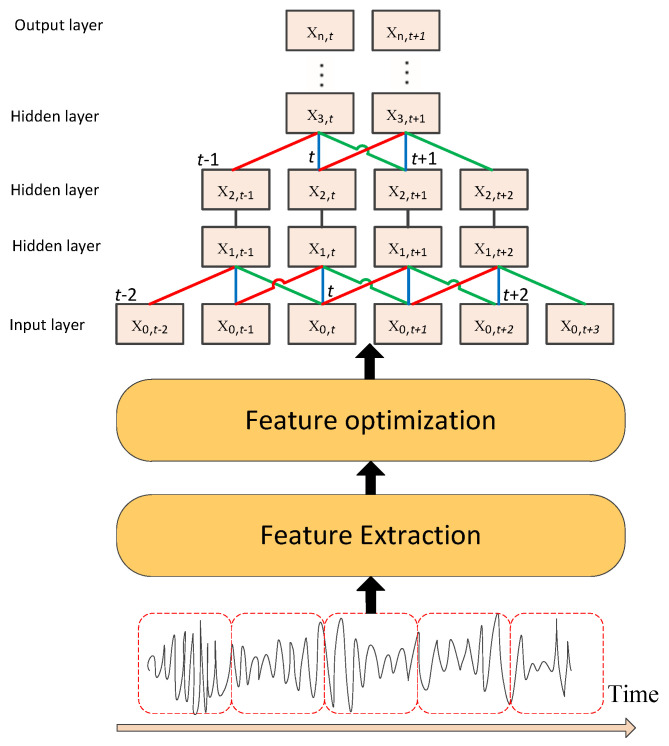
The architecture of Deep Neural Network (DNN) for ship classification.

The overview of the basic framework is shown in Figure 1. It mainly includes two parts, feature extraction and classifier design. For feature extraction, the raw underwater acoustic data is taken as input, then we extract the TF feature with temporal information. On the basis of TF features, filter bank (Fbank) can be obtained by designing different filter banks, which have different weights in different frequency ranges. Furthermore, mel frequency cepstral coefficents (MFCC) can be obtained by using discrete cosine transform (DCT) based on Fbank. Fbank and MFCC are not only common features in traditional target classification, but also widely used in classification tasks based on deep learning. Generally, traditional feature optimization methods such as cepstral mean and variance normalization (CMVN) or L1/L2 normalization [13,14] can improve feature discrimination, but they just refine the features at the mathematical level and ignore the degree of association within the class.

As a classifier, DNN has replaced SVM and other traditional classification frameworks for more and more tasks, and has become a research hotspot. It is composed of an input layer, hidden layer and output layer, and there is no limit on the number of hidden layers. In deep learning, DNNs with two or more hidden layers are used generally. TDNN [15] or long short-term memory (LSTM) [16] are a better choice for samples with temporal information to make final classification.

## 3. Feature Optimization Learning for Ship Classification

### 3.1. Motivation

Different from the air channel, the underwater acoustic channel is affected by temperature and sea conditions easily, which leads to underwater sound speed profile (SSP) change and further affects the transmission channel [17,18]. This causes differences in the received signals from the same target, which seriously affects the performance of the classification model trained by these data.

In classification tasks, obtaining a robust and discriminative representation is crucial for better performance [19,20,21]. Usually, this can be partly achieved by exploiting the TF or DNN-based features [6,22]. However, these features are obtained only from the current signal and the classifiers only focus on finding a decision boundary to separate different classes, without considering the intra-class compactness of the features.

### 3.2. Center Loss

Center loss [11] is proposed to compensate for softmax loss in face verification. It learns a center for the features of each class and meanwhile tries to pull the deep features of the same class close to the corresponding center. Basically, for classification task ${\left\{\left(x_{i},y_{i}\right)\right\}}_{i=1}^{N}$ consisting of N samples $x_{i}$ and their corresponding labels $y_{i}\in \left\{1,2,\ldots ,Y\right\}$. $x_{i}$ is embedding into a new vector $f(x_{i})$ with a DNN [23]. Center loss can be formulated as:

(1) $$L_{c}=\sum_{i=1}^{N}D\left(f(x_{i}),c_{y_{i}}\right),$$

where $c_{y_{i}}$ is the center of class $y_{i}$, function D(·) stands for the distance function. During training, center loss will encourage instances between samples of the same classes to be closer to their learnable class center. However, since the parametric centers are updated at each iteration based on a mini-batch instead of the whole dataset, which is unstable. It usually has to be under the joint supervision of softmax loss during training.

### 3.3. Triplet Loss

Triplet loss is motivated in [12] for image verification. It is expected to make sample $x_{i}^{a}$ closer to all other samples $x_{i}^{p}$ (positive) of the same class than it is to any sample $x_{i}^{n}$ (negative) of other classes. Thus, the function is designed as:

(2) $$L_{tri}=\sum_{i=1}^{N}\left(\left|\right|x_{i}^{a}-x_{i}^{p}{\left|\right|}_{2}^{2}+\alpha -\left|\right|x_{i}^{a}-x_{i}^{n}{\left|\right|}_{2}^{2}\right),\;\forall \left(x_{i}^{a},x_{i}^{p},x_{i}^{n}\right)\in \Omega ,$$

where α is a margin that is enforced between positive and negative pairs. Ω is the set of all possible triplets in the training set. As the training set becomes larger, it is obvious that it will become harder and harder to train the model because the number of triplets will rise exponentially.

### 3.4. The Proposed Feature Optimizer

**Figure 2:**
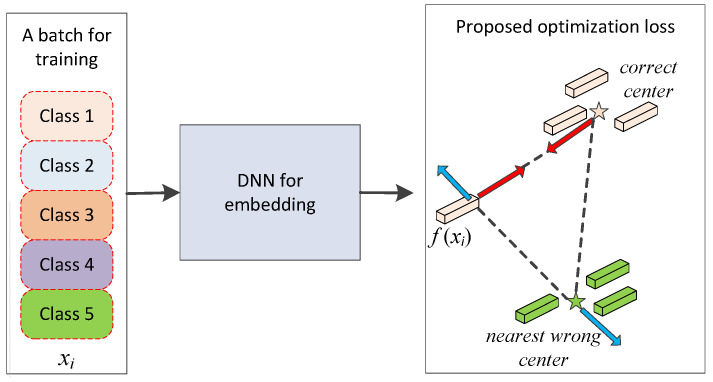
The architecture of the proposed feature optimization.

For solving the problem, the main part of the method we proposed is a feature optimizer, which is trained with a new loss function. We measure the distance between each sample to the class center instead of exhausting all triplets, which will save plenty of training time. It will make model training easier in multiclass classification tasks. The feature optimizer is aimed at making the feature more discriminative by reducing intra-class distance and increasing inter-class distance as illustrated in Figure 2.

As the Figure 2 shows, these feature samples $x_{i}$ are used as inputs of DNN. $f(x_{i})$ is the deep learning feature. Then, it calculates a center for each class by calculating the average of all samples of each class in a minibatch (the five-pointed stars in Figure 2 represent the centers). Based on these centers, for each sample in the minibatch, the distance to each center is measured. Two of these distances are worth paying attention to. One is the distance to its correct center, and the other is to its nearest wrong center. These centers are updated at each iteration. Therefore, for a batch of training data with N samples, the feature optimization $L_{fo}$ loss is defined as:

(3) $$L_{fo}=\sum_{i=1}^{N}\max\left(||f\left(x_{i}\right),\;c_{y_{i}}{\left|\right|}_{2}^{2}-\min_{j\;\neq \;y_{i}}||f\left(x_{i}\right),\;c_{j}{\left|\right|}_{2}^{2}+\alpha ,0\right),$$

where where $c_{y_{i}}$ is the center of class $y_{i}$ and $\left|\right|\cdot {\left|\right|}_{2}^{2}$ represents the Euclidean distance function, *n* is feature dimension:

(4) $${\left||a,\;b|\right|}_{2}^{2}=\sqrt{\sum_{i=1}^{n}{\left(a_{i}-b_{i}\right)}^{2}}.$$

By minimizing feature optimization loss $L_{fo}$, the proposed optimization method will make those samples that are more susceptible to be classified closer to their corresponding right center by a constant α during training. α controls the relative distance between the embedding to its corresponding center and to its nearest wrong center.

### 3.5. Joint Training with Classifier

**Figure 3:**
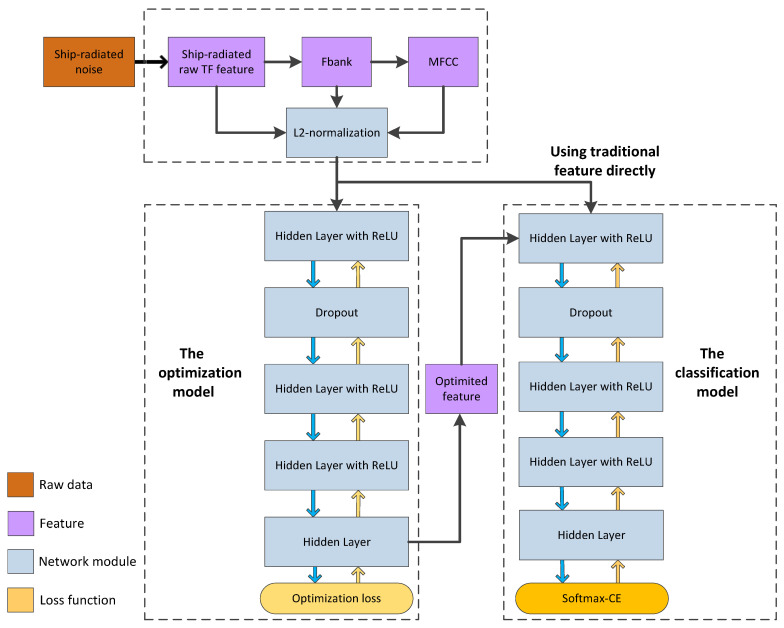
An overview of architecture for joint training. We adopt multilayer DNN as the basic component for achieving representations of ship types, and the proposed optimization loss is used as the supervision loss. In addition, softmax loss could be also combined into the framework for boosting performance. The blue arrow and yellow arrow present forward propagation and backpropagation respectively.

As mentioned above, the parameter of optimization model is updated through a minibatch including different classes data. That means that the centers of each class change in every iteration. A back-end classifier is considered to train with the optimization model jointly. The back-end supervised classifier would serve as a good guide for the direction of optimization and make the learned embedding more discriminative. The classifier would also have a global view to the whole dataset avoiding falling into local optimum during training. These two losses would be combined together to achieve more discriminative embeddings according to our experiments in Section 4, which can be written as:

(5) $$L_{total}=\lambda \;L_{fo}+L_{sotfmax-CE},$$

where λ is a hyper-parameter which controls the trade-off between the feature optimization loss and softmax loss. $L_{sotfmax-CE}$ is defined for minimizing the cross entropy (CE) [24] between estimated target category and the reference target category, given by:

(6) $$L_{sotfmax-CE}=-\frac{1}{N}\sum_{n}\;y_{n}\;ln\;a_{n}+\left(1-y_{n}\right)\;ln\;\left(1-a_{n}\right),$$

where *y* is the excepted output, *y* = {0, 1}, *a* is the actual output between 0 and 1, *n* corresponded to each unit in output layer and *N* is the number of units. We attribute the benefit brought by softmax loss to the fact that the parametric centers of feature optimization are randomly initialized and updated based on the minibatches instead of the whole datasets which might by tricky, while softmax loss would be benefited from seeking better class centers. The whole joint training structure is shown in Figure 3. The left side of the diagram is an optimization model and the right side of the diagram represents a classification model that uses different features. The top of the diagram is the extraction process of traditional features, such as raw ship-radiated spectral feature, Fbank and MFCC. All of these feature will be compared in experiments in Section 4. It is worth mentioning that L2-normalization is adopted in the spectral feature for reducing the difference between different frames in the same recording audio.

## 4. Experiment

### 4.1. Data Description

All data of ship-radiated noise come from a database called ShipsEar [25]. During 2012 and 2013, the sounds of many different classes of ships were recorded on the Spanish Atlantic coast and were included in the ShipsEar database (available at http://atlanttic.uvigo.es/underwaternoise/). The recordings were made with autonomous acoustic digitalHyd SR-1 recorders, manufactured by MarSensing Lda (Faro, Portugal). This compact recorder includes a hydrophone with a nominal sensitivity of −193.5 dB re 1 V/1 upa and a flat response in the 1–28 kHz frequency range. The dataset contains a total of 91 records of 12 vessel types.

As the setup of the author of the dataset, 11 vessel types were merged into four experiment classes (based on vessel size) and one background noise class, which follows the division of data set provider strictly, as follow in Table 1.

**Figure 4:**
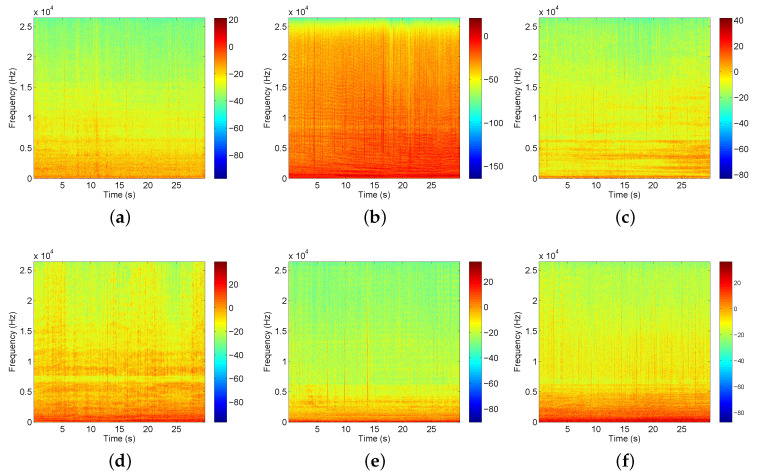
Spectrogram of hydrophone signal for parts of ships. (**a**) background noise; (**b**) motorboats; (**c**) passenger ferries; (**d**) dredger; (**e**) mussel boats; (**f**) ro-ro vessels.

Spectrograms of example signals of different vessels are shown in Figure 4. There are 7503 samples for Class A, 6239 samples for Class B, 7898 samples for Class C, 9122 samples for Class D, 4555 samples for Class E. Each sample lasts approximately 1 s with a sampling frequency of 52,734 Hz. The details of the number of recordings and samples are shown in Table 2.

In our experiment, we divided all recordings into five subsets and a five-fold cross validation procedure was used to test the optimizer and classifier. In order to simulate real application situation, segments in one recording cannot be split into a training dataset and test dataset. The models would be trained by four subsets and the remaining one was used for testing. This procedure was repeated five times so five different models needed to be trained altogether.

### 4.2. Parameters for Feature Extraction

The feature is extracted based on the frequency energy feature. The frame length is about 1 s and the frame shift is about 0.5 s. The bandwidth for 500-Dim Fbank and MFCC feature extraction is within 8 kHz. The Kaldi toolkit [26] is utilized for feature extraction.

### 4.3. Parameters for Feature Optimization Model

The TensorFlow [27] toolkit is utilized for training feature optimization model and DNN-based classification model. The configuration of the DNN-based classification model is that of six layers (one input layer + four hidden layers + one output layer) with 1024 hidden nodes.

The activation function used in our experiment is the Rectified Linear Units (ReLU) [28], which is designed as f(x)=max(0,x). 0.5 dropout is set in first layer to avoid overfitting. The batch size is set to 100, including 20 samples for each class. We sampled the samples cyclically in each class until all samples have been trained. After that, all samples were shuffled once and the next epoch training process starts. L2 normalization is set before features fed into DNN. The embedding dimension is also 500. The initial learning rate for optimizing the model was set to 0.0001. We clip the gradient of center by 0.01 for stable network weight updates.

### 4.4. Parameters for Classification Model

The configuration of DNN-based classification model was similar to the optimization model. The difference is that the normalization function was not used and softmax function was selected to calculate the probability vector in the output layer. The network parameters were uploaded by minimizing the CE between softmax output and the corresponding one-hot vector. The parameters of the model were optimized by back propagation (BP) algorithm with Adam optimizer (β1 = 0.9, β2 = 0.99) [29]. Training was carried out for 300 epochs with a batch size of 100 and a initial learning rate of 0.0001, which decayed with iterations.

### 4.5. Result

#### 4.5.1. Parameter Selection.

**Figure 5:**
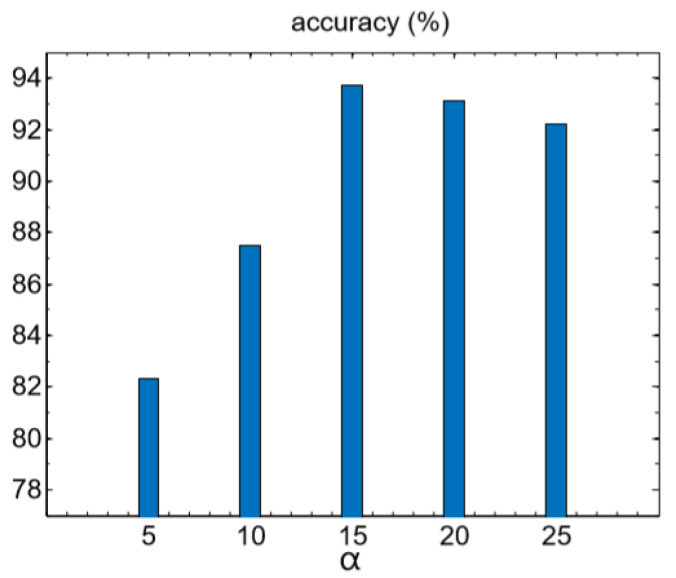
The retrieval performances achieved by varying α.

As indicated by the loss function in Equations (3) and (5), the parameter α and λ would affect all combinations of the losses. λ controls the trade-off between optimization loss and softmax loss. For a wide-ranged values from 0.01 to 1, the model was robust to this parameter. Specially, when λ was set as 0, which means the model was trained using only softmax-CE loss, the performance was the worst. While α controled the relative distance between the embedding to its corresponding center and to its nearest wrong center, it was more sensitive than λ. If it was too small, the optimization effect was weakened and it may cause over-fitting with a too-large value. Obviously, the best parameter selection was different in different datasets. An analysis was necessary to determine the best value of α and λ. We tried different λ from 0.01 to 1 while observing the change of loss function at the same time. We found it had little effect on the performance of the final model. λ is fixed to be 0.1, and then set α to be 5–25, respectively. As the Figure 5 shows, we set α to be 15 in default for following experiments.

#### 4.5.2. Experiment Results and Discussion.

**Figure 6:**
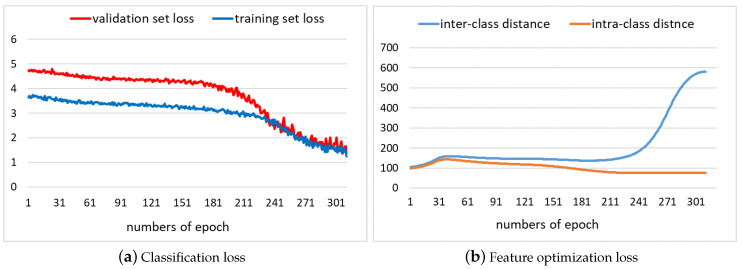
The classification loss curves and feature optimization loss curves with iterations.

The convergent tendency of our proposed loss function with optimization method is shown in Figure 6a while Fbank was chosen as the basic input feature. It is observed that the classification model trained by optimized feature can converge normally. Figure 6b shows changes of the intra-class distance and the inter-class distance respectively, which means the optimization model had the ability to pull samples of the same class together as much as possible, and push samples of different class away. Thus, the classification model would be easier to use for distinguishing these embeddings.

The basic input and output of the model were all based on frames. We calculated the frame accuracy of recordings by counting the posterior probability of output of the model. If the classification results of most frames from a recording were correct, we consider that the classification of the recording was correct, and then calculate the utterance accuracy. All experimental results after this subsection were based on utterance accuracy.

Table 3 summarizes the results of using raw TF feature and handcrafted traditional features such as Fbank and MFCC in comparison with DNN classifier (with or without optimization method) and the baseline proposed by Santos-Dominguez [25], who is the provider of Shipsear. We notice that Fbank with optimization method obtains the best result in our experiment. It indicates it is beneficial to increase the weight of the low frequency part in the feature, but more complex spectrum transformations (such as discrete cosine transform) may make it worse. For all three different features, the utterance accuracy is significantly improved while feature optimization method is used. Relative improvements of 13.3%, 10.5%, and 7.4% were obtained on raw TF feature, Fbank, and MFCC, respectively. Therefore, it can be concluded that the optimization method based on feature distance has stable improvement for multiple features.

Furthermore, it is clear from Table 3 that the method we proposed has an absolute 9% increase compared with Santos-Dominguez’s method [25]. Compared with the method proposed by Santos-Dominguez, we found that our classifier, trained by the optimized feature, can achieve higher utterance accuracy than the original method in classifying all four vessel classes while maintaining 100% accuracy in detecting vessel presence.

Table 4 shows the confusion matrix for this experiment using optimized Fbank. It is obvious that there is no confusion between background noise and the other types of ships. More confusion occurs in Class A, B and C. One possibility is that the displacement of vessels in Class D is much greater than that of Class A,B,C, which makes it easier to distinguish the ship-radiated noise in Class D.

#### 4.5.3. Comparison with Other Loss Functions and Optimization Models.

To validate our proposed loss, we extended experiments on other losses, including center loss with softmax (it is hard to train with center loss only) and triplet loss using Fbank. Since the training time of using all triplets is much higher than that of the other two methods, which cannot be completed in limited time, we select 1000 positive samples and 1000 negative samples randomly in every model training. As can be seen in Table 5, our proposed loss function get the best recognition effect. On the other hand, we add time delay −1,0,10−1,0,10−1,0,1 to the current DNN, which means previous and next frames are spliced to current frame in the input layer, the second hidden layer and the forth hidden layer. TDNN was used as optimization model for comparison of different loss functions. It can be seen that while the optimization model was changed from DNN to TDNN, the loss function we proposed can still achieve robust improvement compared with the previous loss functions.

#### 4.5.4. Visualization of Learned Representations.

**Figure 7:**
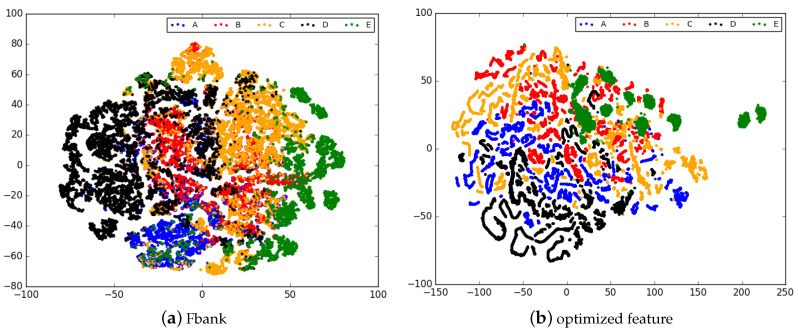
Visualization of Fbank and optimized feature.

We adopt t-SNE [30] to visualize the optimized features of the samples from our test set. As is shown in Figure 7, compared with the Fbank, the learned embeddings using optimization method make clusters of the same class more compact and different ones more separated at the same time. This demonstrates that better underlying representations for classification task can be obtained using our proposed joint optimization loss.

## 5. Conclusions

In this paper, we focus on a feature optimization method and propose an optimization loss function for ships classification tasks and obtain the best result 84% on the standard five-class data set. This loss function can optimize features directly by minimizing the intra-class distance while also maximizing the inter-class distance at the same time through a DNN-based network. As a result, the learned embeddings are more robust and discriminative, thus more appropriate for the classification task.

## Figures and Tables

**Table 1:** The 11 vessel types were merged into four experiment classes and one background noise class.

Class A	fishing boats, trawlwes, mussel boats, tugboats and dredger
Class B	motorboats, pilot boats and sailboats
Class C	passenger ferries
Class D	ocean liners and ro-ro vessels
Class E	background noise recordings

**Table 2:** Database composition of part types of ships in ShipsEar.

		Class				
		A	B	C	D	E
Database material	number of recordings	17	19	17	10	12
	number of samples	7503	6239	7898	9122	4555

**Table 3:** The accuracy comparison on Shipsear dataset among Santos–Dominguez’s, DNN, and op+DNN, op+DNN means using optimization model and DNN classification model.

Method	Basic Feature	Class A	Class B	Class C	Class D	Class E	Average
Santos-Dominguez’s [25]		0.625	0.800	0.764	0.555	1.000	0.754
DNN	raw TF feature	0.529	0.579	0.588	0.500	0.833	0.600
	Fbank	0.706	0.684	0.647	0.800	1.000	0.760
	MFCC	0.706	0.684	0.647	0.700	0.900	0.733
op+DNN	raw TF feature	0.588	0.632	0.647	0.600	1.000	0.680
	Fbank	**0.824**	**0.789**	**0.765**	**0.900**	1.000	**0.840**
	MFCC	0.749	0.737	0.706	0.800	1.000	0.787

**Table 4:** Confusion matrix for the performance of our proposed method. Each cell shows the number and percentage of vessels for the classes in the rows classified in the classes in the columns. The diagonal indicates the number of correctly classified utterance. Each utterance is an audio file containing a recording of a single vessel. Classes A–D refer to four vessel classes and class E refers to background noise.

		Predicted Sound				
		A	B	C	D	E
Actual sound	A	14 (82.4%)	2 (11.8%)	0 (0%)	1 (5.9%)	0(0%)
	B	1 (5.3%)	15 (78.9%)	2 (10.5%)	1 (5.3%)	0 (0%)
	C	1 (5.9%)	3 (17.6%)	13 (76.5%)	0 (0%)	0 (0%)
	D	0 (0%)	1 (10%)	0 (0%)	9 (90%)	0 (0%)
	E	0 (0%)	0 (0%)	0 (0%)	0 (0%)	12 (100%)

**Table 5:** Accuracy comparison with different optimization models and different loss functions.

Optimization Model	Loss Function	Accuracy %
DNN	Center loss + Softmax	0.803
	Triplet loss + softmax	0.786
	Proposed loss function	0.840
TDNN	Center loss + Softmax	0.813
	Triplet loss + softmax	0.786
	Proposed loss function	0.840
